# PhyreStorm: A Web Server for Fast Structural Searches Against the PDB

**DOI:** 10.1016/j.jmb.2015.10.017

**Published:** 2015-10-27

**Authors:** Stefans Mezulis, Michael J.E. Sternberg, Lawrence A. Kelley

**Affiliations:** **Structural Bioinformatics Group**, Imperial College London, London SW7 2AZ, United Kingdom

**Keywords:** structural alignment, structural search, one-vs-many, protein, TM-align

## Abstract

The identification of structurally similar proteins can provide a range of biological insights, and accordingly, the alignment of a query protein to a database of experimentally determined protein structures is a technique commonly used in the fields of structural and evolutionary biology. The PhyreStorm Web server has been designed to provide comprehensive, up-to-date and rapid structural comparisons against the Protein Data Bank (PDB) combined with a rich and intuitive user interface. It is intended that this facility will enable biologists inexpert in bioinformatics access to a powerful tool for exploring protein structure relationships beyond what can be achieved by sequence analysis alone. By partitioning the PDB into similar structures, PhyreStorm is able to quickly discard the majority of structures that cannot possibly align well to a query protein, reducing the number of alignments required by an order of magnitude. PhyreStorm is capable of finding 93 ± 2% of all highly similar (TM-score > 0.7) structures in the PDB for each query structure, usually in less than 60 s. PhyreStorm is available at http://www.sbg.bio.ic.ac.uk/phyrestorm/.

## Introduction

Structurally aligning a protein against a database of known structures is a widely used technique in the biological sciences. The results of one-versus-many structural alignments have been used to identify novel conformations or folds [1] and to refine probe structures used for molecular replacement [2], and these are commonly used for structural analysis of newly resolved structures. In addition, it is well known that protein structure is conserved across larger timescales than sequence. Three-dimensional alignment can therefore provide significant clues regarding protein function and evolution not detectable by sequence similarity alone.

When investigating the structural relationships between a protein of interest and the database of known structures, several features are of key importance: coverage, accuracy, speed and ease of use. Of these, coverage, accuracy and speed are strongly linked: because of the ever-increasing number of protein structures contained within the Protein Data Bank (PDB) and the computational expense of a structural alignment, scanning a query structure against the PDB can take several CPU-days (CPU, *c*entral *p*rocessing *u*nit). For example, aligning two structures with TM-align takes approximately 0.5 s [3] and the PDB contains approximately 300,000 chains, requiring 43 CPU-hours.

For one-off searches, such as might be performed with a newly determined structure, the time required for a search is relatively unimportant. However, an important use case of an automated alignment server is exploratory or hypothesis-driven searching. For example, our Phyre2 protein structure prediction server [4] produces tens of models for a given protein sequence. The ability to search these models against the PDB in order to illuminate functional or structural relationships is commonly requested by our users. For this to be of use, results must be found quickly. Similarly, the identification of structural similarity of proteins in a macromolecular complex to other proteins could require repeated database searches. If a search is to be completed in less than a minute using TM-align, approximately 2500 CPUs would be required, well beyond the capacity of a typical academic compute farm. Therefore, tools must either increase the speed of the alignment algorithms or reduce the coverage of the structural database.

Of the existing one-versus-many structural alignment tools, probably the most well known are Dali (distance matrix alignment program) [5], FATCAT (flexible structure alignment by chaining aligned fragment pairs with twists) [6], VAST (vector alignment search tool) [7], SSM (secondary structure matching) [8] and the CATH Database search tool [9,10]. Each of these tools uses a different method to align structures. Dali builds a residue-residue distance matrix for each protein and aligns these matrices, FATCAT connects aligned fragment pairs using dynamic programming and both VAST and SSM align vectors pointing along secondary structure elements. CATH uses a hybrid approach combining secondary structure matching and dynamic programming.

VAST and SSM use a simplified representation of protein structure, offering a significant increase in speed when compared to Dali and FATCAT. This allows VAST and SSM to search the entire PDB in a reasonable time by simply aligning the query to each structure in turn. During testing, described in the supplementary information, VAST took approximately 2 h to process each query. SSM takes approximately 30 s for each query at the default settings, increasing to approximately half an hour when set to find less similar structures.

The alignment algorithms used by Dali and FATCAT are more computationally expensive than those used by SSM and VAST. Thus, to maintain a high speed, both Dali and FATCAT compromise the coverage of the PDB. The largest database that can be searched by the FATCAT server is the PDB clustered to 90% sequence identity (PDB90). Dali uses a bespoke database and search method that is in theory capable of finding all similar structures; by design, however, Dali will only ever return a maximum of 1000 alignments. For each query, Dali takes approximately an hour to finish. In our trials, we were only able to obtain results from Dali and SSM, and thus, only data from these systems are included in the supplementary information. Of the 10 query structures used for benchmarking Dali and SSM, PhyreStorm finds at least as many high-quality structures in all but one case, for which PhyreStorm misses two structures found by SSM.

Reducing the size of the structure database mitigates the computational burden of each search but might well miss structures that imply interesting functional and evolutionary relationships. This problem becomes especially pronounced when the structural database is reduced by clustering structures by sequence and choosing representatives for each cluster. This method picks an arbitrary structure from an ensemble of conformations with no guarantee of choosing a representative structure, an especially egregious problem in the case of structures with alternative conformations—“open” and “closed”, for example—for which one is arbitrarily discarded.

In this paper, we introduce PhyreStorm (Phyre: searching topology by rapid matching), a tool for fast and accurate structural alignment against the entire PDB. This is a standalone Web server and will be an additional tool in the Phyre2 [4] protein modeling portal. For a given query structure, PhyreStorm aims to identify *every* similar structure—and *only* the similar structures—and to build high-quality alignments for each.

PhyreStorm avoids compromising either the database or the alignment quality by using a hierarchical database in which structures are grouped by structural, rather than sequence, similarity. When a query is aligned to the database, the representatives of each cluster are aligned to the query and all clusters with poorly matching representatives are discarded. Next, the members of each remaining cluster are aligned to the query (see The PhyreStorm database). This reduces the number of alignments that must be performed for each search and allows the use of a high-quality alignment algorithm (see TM-align for details of the alignment algorithm used by PhyreStorm).

## Search Algorithm and Database

To avoid compromising search speed, alignment accuracy or database coverage, PhyreStorm takes advantage of structural relationships made available by the Research Collaboratory for Structural Bioinformatics (RCSB) [11] to avoid processing structures that cannot possibly be similar to the query structure. The remaining alignments are processed in parallel, taking advantage of unused capacity in our existing compute farm.

### TM-align

PhyreStorm uses the well-established alignment method TM-align [3], used throughout the protein modeling community to assess the quality of models produced by protein structure prediction servers.

TM-align uses dynamic programming to directly align the C^α^ atoms of two proteins. An alignment is scored by the TM-score:

(1) $$\text{TM-score}=\frac{1}{L_{q}}\sum_{i}^{L_{a}}\frac{1}{1+{\left(\frac{d_{i}}{d_{0}(L_{q})}\right)}^{2}},$$

where *L_q_* and *L_a_* are the lengths (in residues) of the query protein and alignment respectively, and *d_i_* is the distance in angstroms (Å) between the C^α^ atoms of each aligned residue pair. The parameter *d_0_* is a normalization parameter that depends on the size of the query protein and removes the power-law dependence on protein length often found in alignment scoring functions. Compared to GDT or MaxSub, TM-score produces rankings that agree more consistently with rankings by humans [12].

A useful property of the TM-score is that it can provide a good predictor of whether a protein is in the same fold [13]: in general, proteins with a TM-score above 0.5 will be of approximately the same fold.

### The PhyreStorm database

In order to reduce the number of alignments necessary when scanning a structure, PhyreStorm begins by segregating the PDB into structurally similar clusters. The RCSB makes available a database of structural similarities between all structures in a subset of the PDB [11]. The subset from which these relationships are derived is the PDB clustered to 40% sequence identity (PDB40), and the database consists of pairwise alignments generated using the FATCAT algorithm between all structures in PDB40. Each structure in PDB40 is separated into domains. If the structure is present in SCOP (structural classification of proteins) 1.75 [14], then the SCOP domain definitions are used; otherwise, the structure is automatically split using PDP (protein domain parser) [15].

We used the TM-score (as calculated by FATCAT) between each pair of domains in PDB40 as a similarity metric to group PDB40 into structurally similar clusters. Clusters were built by EzClust, an in-house tool using an agglomerative hierarchical clustering algorithm [16]. The clusters were chosen such that the average TM-score between all pairs of structures within the cluster is no less than 0.5, indicating a similar fold [13].

Next, any structures that were pruned from the PDB to make PDB40 are added. This is performed using the sequence cluster definitions provided by the RCSB^†,‡^. Each structural cluster *s_i_* found by EzClust is examined in turn. Each member *s_ij_* of *s_i_* is then examined. If *s_ij_* is in a sequence cluster *S_k_* provided by the RCSB, then all members of *S_k_* are added to *s_i_*. Each member of *S_k_* is assumed to have the same structural relationships as *s_ij_*. A representative is then elected for each cluster by finding the structure with the maximum average similarity with all other structures in the cluster. Finally, all sequence clusters that contain structures that have not been added to the database are added as separate clusters.

The database is updated each week in step with the PDB. If the RCSB has updated the PDB40-*versus*-PDB40 results, then a new database is built. Otherwise, the new PDB structures are scanned against the database using PhyreStorm and added to each matching cluster.

**Fig. 1:**
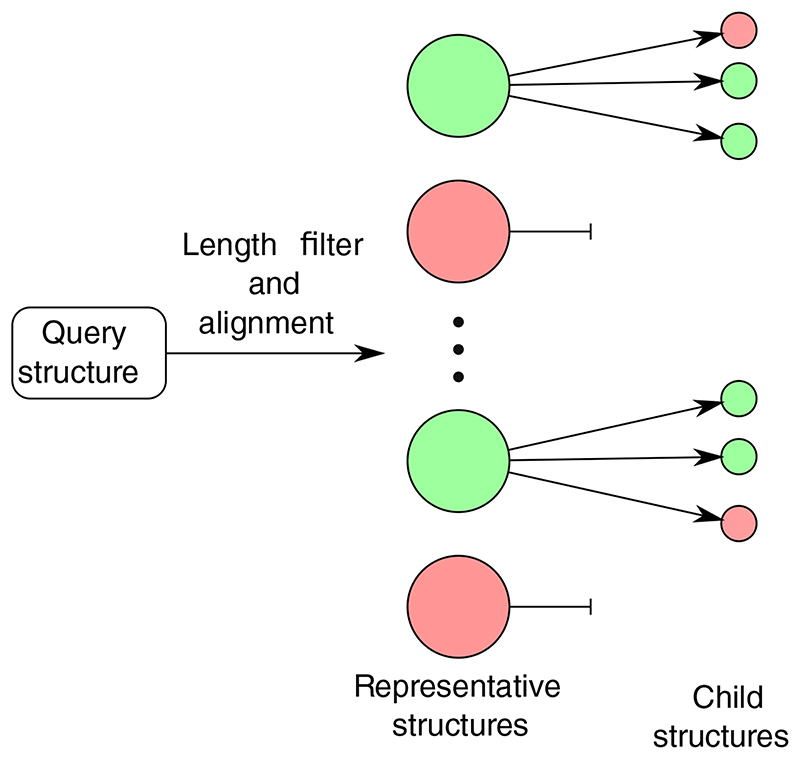
Searching the PhyreStorm database. First, representatives are discarded if they are below a minimum length, as they cannot possibly score well. Then, the query structure is aligned against all remaining representatives. Representatives with a poor score (shown in red) are discarded. Clusters with a high-scoring representative (green) are expanded and the query is aligned against all the children. All children with a score above the threshold are reported, and low-scoring children are discarded.

To search the database, we align a query structure against the representatives of each cluster (Fig. 1). All representatives with a TM-score below 0.5 are discarded, as the children of that cluster cannot match well with the query. Since TM-score is normalized by the length of the query structure, representatives less than half the length of the query may be immediately discarded without alignment; this reduces the number of alignments required for large structures, effectively offsetting the increased time required to align large structures. Finally, after finding the clusters with representatives that match well against the query structure, each of the other members of the clusters is aligned with the query.

When aligning a query against a protein in the database, PhyreStorm expands domains to entire chains. That is, if a structure is labeled as domain *i* of chain *x* in a protein, the alignment is performed against the whole of chain *x*. This allows multi-domain queries to align well with multi-domain proteins, and this eliminates the effects of the automatic domain assignment. See Discussion and conclusion for a discussion regarding multi-domain queries and templates.

## Results

To determine the impact of clustering on coverage, we processed a benchmarking set of 100 representative protein domains. Only single domains were used because determining what should be classed as structurally similar match becomes difficult for multi-domain proteins; see Discussion and conclusion for a discussion on possible solutions to this problem. The benchmarking set was chosen from SCOPe v2.04 [17] such that no more than two proteins are from a single superfamily in order to determine performance across a wide range of topologies.

**Fig. 2:**
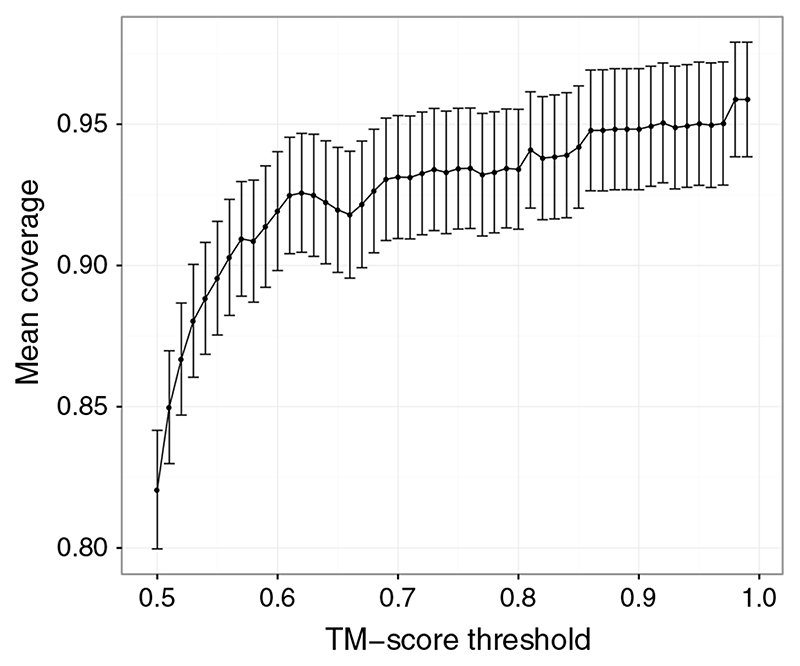
Average coverage for 100 query structures. Coverage is calculated by n_PS_(TM > x)/n_PDB_(TM > x), where n_PS_ and n_PDB_ are, respectively, the number of structures found using PhyreStorm and by searching the entire PDB. The error bars show the standard error.

The gold standard against which PhyreStorm was compared was an alignment against every chain in the PDB. Alignments were classed as missing if a structure was found in the PDB with a TM-score above 0.5 but was not found by PhyreStorm. These results are shown in Fig. 2.

Figure 2 shows that PhyreStorm performs well at finding structures very similar to the query, with performance dropping slightly at lower TM-score thresholds. This is because of the organization of the database; similar structures are grouped together at a TM-score threshold of 0.5 such that the average TM-score between structures in a cluster is no less than 0.5. If a query structure matches a cluster only tentatively, it is likely to match only a portion of the structures contained within that cluster and there is no guarantee that the matching set of structures includes the cluster representative. If the query does not match the cluster representative, then the children of the cluster will not be aligned and the matching children cannot be found.

Comparison to other one-versus-many alignment services is complicated by the different alignment algorithms and scoring methods used by each. Distilling the alignment of two complex three-dimensional structures into a single number is inevitably difficult, and there is some disagreement in the literature as to the accuracy of various alignment methods and how to compare different methods [18–21]. A comparison of PhyreStorm with other tools is given in the supplementary information. To summarize, PhyreStorm finds at least as many highly similar structures as Dali and SSM and many more structures of intermediate similarity.

### Interface

The PhyreStorm interface is designed for ease of use by non-experts. To start PhyreStorm, users may upload a structure or enter a PDB code. It will soon be possible to submit models from the results page of the Phyre2 structure prediction server [4]. If a PDB code is entered without a chain identifier, an interactive view of the PDB structure is shown, allowing the user to select a chain. If the chain is set to “*” or the “Merge chains” option is selected from the interactive view, then all chains in the PDB file will be merged. No email address is required for submission.

When a structure is submitted, the user may select the required degree of similarity such that only structures more similar than this value will be displayed. The default value of TM > 0.6 finds structures with a relatively high degree of similarity, and it is likely to be useful for exploring functional and evolutionary relationships. Lowering the threshold value to 0.5 will find many more structures of a similar fold as the query, finding results more useful for exploring the conformation space occupied by the query structure. The similarity threshold cannot be lowered further, as this would return many spurious results.

**Fig. 3:**
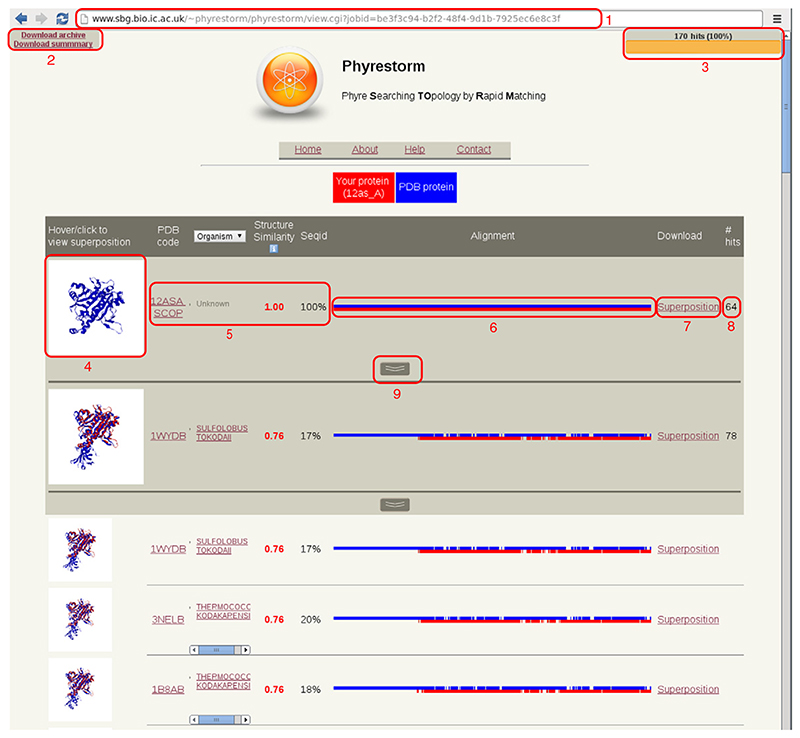
The PhyreStorm interface, displaying the following elements for the query structure 12as (chain A): (1) unique URL, (2) archive and summary download links, (3) job progress, (4) static image of the superimposed structures, (5) alignment information and scores, (6) one-dimensional representation of the alignment, (7) download link for superposition coordinate file, (8) number of members of this cluster that have aligned well with the query and (9) button to expand the members of this cluster.

Once a search has been started, the user is immediately redirected to the results page (identified by a unique URL by which results may be shared), shown in Fig. 3. The Web browser maintains a connection to the PhyreStorm server, allowing alignments to appear as soon as they are processed.

Each row of the results table shows the results for an alignment with the representative of a cluster of similar structures. This provides a quick idea of the different conformations populating the fold occupied by the query structure. The user may expand a cluster to display the ensemble of structures closest to the representative structure. The results page of PhyreStorm is roughly analogous to the “topology” pages of CATH [9]: all results found by PhyreStorm are of similar topology, and each cluster contains highly similar structures.

For every result, one-dimensional and three-dimensional representations of the alignment are displayed. If a user hovers over the image of the three-dimensional superposition, a pop-out box will be displayed with a larger version of the image. If the user clicks the image, an interactive view of the superposition will be rendered using 3Dmol [22]. A click on the one-dimensional representation will display a detailed sequence alignment with the option to download the alignment in FASTA format or the original output from TM-align.

Between the two alignments are the PDB identifier, metadata from the PDB entry, structural similarity score and sequence identity. If the target structure is a SCOP [14] domain, a link is provided to SCOPe [17]; if the target is a domain built using PDP [15] by the RCSB, a link is provided to the RCSB structural summary. The protein metadata by default display the PDB title, but a drop-down box in the table header provides the option to switch to the PDB keywords or organism data. If the organism data are provided by the PDB entry, it links to the National Center for Biotechnology Information taxonomy browser [23].

To the right of the one-dimensional alignment is a link to download a coordinate file containing the superposition. This file is a rasmol [24] script but can be displayed without modification in PyMOL or other visualization tools.

Finally, when the alignment is complete, a link to download a (bzipped) archive of results and a link to a parseable summary appear in the top left corner.

## Discussion and Conclusion

Determining which alignments are significant for multi-domain proteins is a difficult task, and it depends on the requirements of the user. It is not clear whether results that align well to a single domain should be considered significant, if results that contain similar domains in different orientations should be considered significant or whether only results for which all domains align well and in the same orientation should be considered significant.

For example, consider a protein of length *L* with *n* domains each of length *l_1_*, *l_2_*,…*l_n_*. By examining Eq. (1), it can be seen that the maximum TM-score that can be obtained by matching well to the single domain *i* is *l_i_/L*. Therefore, high-quality matches to a single-domain protein will not be considered significant if *l_i_/L* is less than the required similarity threshold.

The PDB40-*versus*-PDB40 data supplied by the RCSB, on which the PhyreStorm database is built, are based on domain-domain alignments. If a protein has an entry in SCOP (version 1.75 at the time of this writing) [14], then the SCOP domain definitions are used; otherwise, the PDP [15] is used to automatically split each protein into domains. After the PDB40-*versus*-PDB40 alignments have been used to build clusters, each cluster will only contain single domains. After sequence homologues are added (as described in Search algorithm and database), the cluster may contain some multi-domain proteins, but the single-domain proteins are more likely to be elected as representatives because of the higher internal similarity. This could lead to PhyreStorm missing some structures that would align well to a multi-domain query if they are “hidden” behind a single-domain representative (see Supplementary Fig. 3 for more information). This problem is mitigated in PhyreStorm by expanding single domains into the entire protein chain (see Supplementary Fig. 3).

To avoid these problems, we encourage Phyre-Storm users to split their query proteins into individual domains. A planned feature is to provide an interface with a structure and sequence view of the query protein with domains cut at the best guess available from automated software. The user will be able to adjust these domains and submit each domain to PhyreStorm for search. Upon completion of the search, PhyreStorm will provide the ability to apply set operations to the results (such as the intersection of all results containing domains 1 and 2, or all results containing domain 1 and all results containing domain 2). We are currently awaiting user feedback regarding the design of this feature.

A similar issue can occur if the query protein consists of multiple chains, possibly generated using the “merge chains” option of PhyreStorm. In this case, the problem becomes more severe: because the PhyreStorm database is composed of single chains, the length threshold is significantly less likely to be met.

In some cases, the same problem can occur when the query protein contains a common substructure. Consider a query protein composed of a common substructure *A* and a unique substructure *B*. In this case, the best-matching clusters in the PhyreStorm database are likely to be represented by structures similar to A, as *B* is unique to the query. If the length of *A* is less than the TM-score threshold, then matches between the query protein and substructures similar to *A* will not be considered significant, and PhyreStorm will return no results. This is the desired behavior if the goal is to find structures similar to the entire query, as none exists, but it is unintuitive to a user who knows that many structures that share substructure *A* exist.

The PhyreStorm Web server is a fast, accurate and comprehensive tool for aligning a protein structure against the PDB. By discarding structures that cannot align well to the query structure, PhyreStorm achieves an order of magnitude reduction in the number of alignments required for each search, allowing a slow but sensitive gold standard alignment algorithm to be used. It is intended that the high coverage and speed of PhyreStorm, combined with the rich and intuitive user interface, will provide biologists unfamiliar with bioinformatics with the necessary tools to explore protein structure relationships beyond what is currently possible.

## Supplementary Material

SI Supplementary data to this article can be found online at http://dx.doi.org/10.1016/j.jmb.2015.10.017.

## Footnotes

†
http://www.rcsb.org/pdb/statistics/clusterStatistics.do.

## Abbreviations used

PDB: Protein Data Bank
RCSB: Research Collaboratory for Structural Bioinformatics
