# Supplementary material for: PhyreStorm: A Web Server for Fast Structural Searches Against the PDB
Source: J Mol Biol. Author manuscript; Available in PMC 2021 Jun 10. (PMC7610957; doi:10.1016/j.jmb.2015.10.017)
Supplement: SI — Supplementary data to this article can be found online at http://dx.doi.org/10.1016/j.jmb.2015.10.017. [file EMS127161-supplement-SI.pdf]

PhyreStorm: a web server for fast structural searches against the PDB
Supplementary Information

Stefans Mezulis[a,*], Michael J. E. Sternberg[a], Lawrence A. Kelley[a]

[a]*Structural bioinformatics group, Imperial College London, London, SW7 2AZ, UK*

1. Comparison of PhyreStorm with other tools

In section 3 of the main text, the coverage of PhyreStorm is examined with respect to all chains in the PDB with a TM score of greater than 0.5. Here, we examine the performance of PhyreStorm when compared to alternative tools.

To avoid placing undue load on the servers, the benchmarking set described in the main text was reduced to 10 randomly chosen proteins (listed in "subset_for_external_webservers.csv" in the supplementary information). Each of these proteins was run through VAST, Dali and SSM. Model quality was evaluated using GDT_TS[1], which was chosen because it is a commonly-used measure of similarity that is not directly optimised by any of the methods tested here. Examples of structures at different GDT_TS thresholds are shown in figure 2.

To estimate the GDT_TS score of each model, the FASTA alignments given by each method were used to build a protein model in a manner identical to that of a homology modelling server. The program MaxCluster[2][1] was then used to calculate the GDT_TS score of each model. The rotation matrix produced by MaxCluster is generated by optimising the MaxSub score and is therefore not necessarily identical to the rotation matrix produced by the servers; however, the difference in GDT_TS score between superpositions calculated in this way and superpositions generated from the server is minimal, because each model only contains the aligned residues as calculated by each server.

Only comparisons with Dali and SSM are presented here. This is because VAST does not provide any bulk download services, and results are paginated in groups of 60. Thus, downloading every alignment requires a web crawler and several thousand HTTP requests. Results from FATCAT and other alignment tools such as the CATH database structural search[3] could not be included as, at the time of this writing (July 30th, 2015), no query structures were successfully processed by the servers.

*Corresponding author
[1]http://www.sbg.bio.ic.ac.uk/~maxcluster/

Dali provides no adjustable parameters, and so the results presented here are all that can be obtained. The version of Dali used to generate these results was DaliLite 3.3, used with the "-Q" option.

SSM (`http://www.ebi.ac.uk/msd-srv/ssm/`, accessed on July 27th, 2015) offers the option to lower the threshold for whether an alignment will be considered "similar". However, lowering this threshold often resulted in an internal server error and so the default settings were used. When SSM finds no matches it suggests using a lower similarity threshold; in these cases, the suggested values were used.

Comparison of results is further complicated by the presence of obsolete structures in the databases used by each method, and so the results presented in figure 1 are filtered to exclude obsolete structures. Some structures that have been declared obsolete by the PDB have been superseded by new PDB codes that are not available in PDB format. All of the tested tools require files in the PDB format, and so superseded files that appear in the results when the superseding file does not are retained; this prevents overcounting due to duplicate structures, but allows a structurally correct result to be kept.

Not all of the results from each server could be obtained at the same time. To avoid bias due to extra structures deposited in the PDB in the intervening time, the results from each server were filtered to a common subset. The subset chosen here was the PDB at the time the benchmarking set was first chosen (March 11th, 2015, supplied in the supplementary file "bc-40.csv").

It can be seen from figure 1 that PhyreStorm (at a similarity threshold of 0.5) finds at least as many high quality (GDT > 60) structures as Dali and SSM. At lower similarity thresholds, PhyreStorm finds many more structures.

In no cases does Dali find more results (with a GDT score above 60) than PhyreStorm. For the query d4gqhq_, SSM finds 18 more structures with a GDT score above 60 than PhyreStorm. This is because PhyreStorm misses one structure that is a homo 17-mer.

2. Parallel processing architecture

Clustering the PDB as described in the main text significantly reduces the number of alignments required to find all similar structures. However, several thousand alignments must still be performed. To provide a fast user experience, PhyreStorm runs in parallel on our existing compute farm.

PhyreStorm bypasses the batch system commonly used in academic compute farms. Each individual alignment is a small task, and so by running a low-priority daemon on each compute node PhyreStorm effectively runs "in the gaps" between jobs. This increases the utilisation of our resources by allowing useful work during otherwise idle times when nodes are performing I/O or waiting for more jobs to be scheduled.

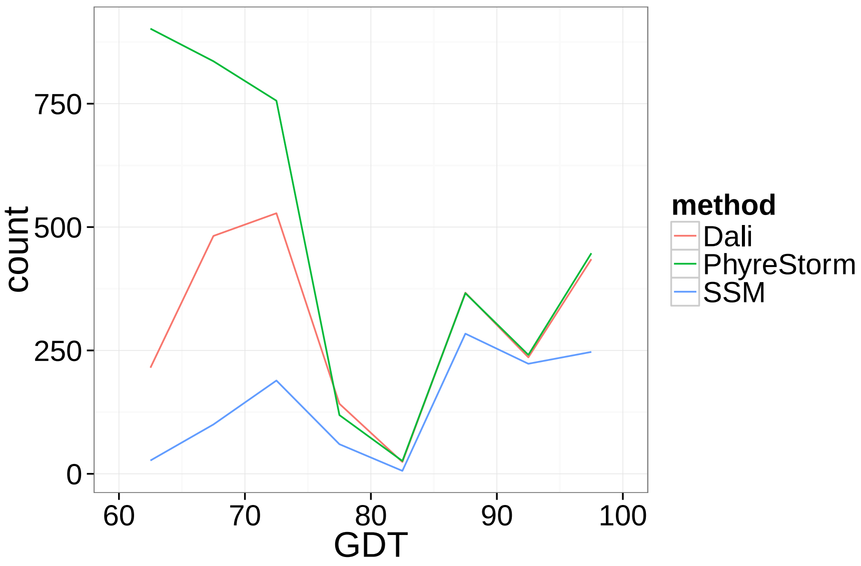

Figure 1: Frequency polygons showing the number of structures found with a certain GDT (Global Distance Test). The bin width is 5 GDT points.

3. Multi-domain queries

Figure 3 illustrates the problems encountered when searching multi-domain query proteins against the PhyreStorm database, and shows the multi-domain query AB searched against the PhyreStorm database. If the query is not split into separate domains, as in figure 3a, then AB will be aligned to the cluster representative A_R. A_R will align well, but if $L_{A_R} < t_{TM} L_{AB}$ (where L_{A_R} and L_{AB} are lengths of A_R and AB, and t_{TM} is the minimum TM-score threshold for an alignment to be considered significant) then the cluster will not be expanded and the best-matching structure in the database, $A''B'$, will be missed.

PhyreStorm expands single domains into the entire protein chain. If A_R is a single domain excised from a larger chain, then AB will be aligned against the whole chain containing A_R. If the whole chain matches well to AB, then the cluster will match well.

If the query is split into domains, as in figure 3b, then domain A will align well to the representative A_R of cluster c. Cluster c will then be expanded and A will be aligned to $A'C$ and $A''B'$, each of which will align well.

[1] A. Zemla, LGA: a method for finding 3d similarities in protein structures, Nucleic Acids Research 31 (13) (2003) 3370–3374.
URL http://www.ncbi.nlm.nih.gov/pmc/articles/PMC168977/

[2] A. D. Herbert, Protein structure prediction using fragment libraries, Ph.D. thesis, Imperial College London (Feb. 2007).

[3] C. A. Orengo, A. Michie, S. Jones, D. T. Jones, M. Swindells, J. M. Thornton, CATH – a hierarchic classification of protein domain structures, Structure 5 (8) (1997) 1093–1109. doi:10.1016/S0969-2126(97)00260-8. URL http://www.sciencedirect.com/science/article/pii/S0969212697002608

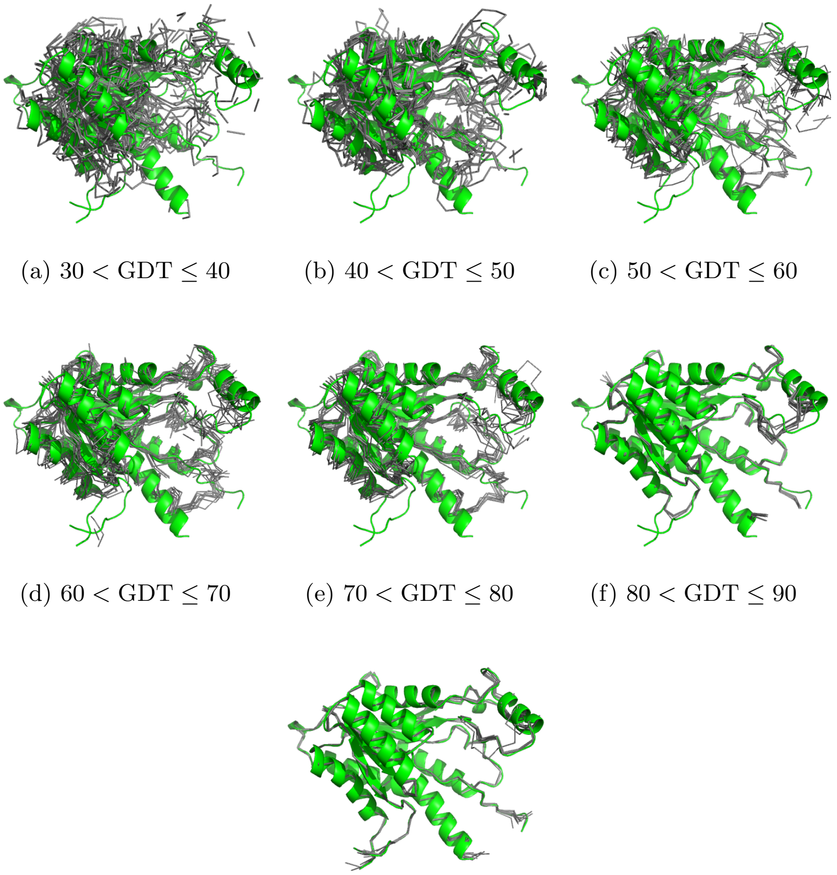

(a) $30 < \text{GDT} \leq 40$ (b) $40 < \text{GDT} \leq 50$ (c) $50 < \text{GDT} \leq 60$

(d) $60 < \text{GDT} \leq 70$ (e) $70 < \text{GDT} \leq 80$ (f) $80 < \text{GDT} \leq 90$

(g) $90 < \text{GDT} \leq 100$

Figure 2: Ensembles of structures with varying GDT scores. The cartoon structure is the SCOP domain `d2bfob_`. Each cartoon structure has 10 structures (found by PhyreStorm) aligned to it using TM-align. Images generated using the PyMOL Molecular Graphics System.

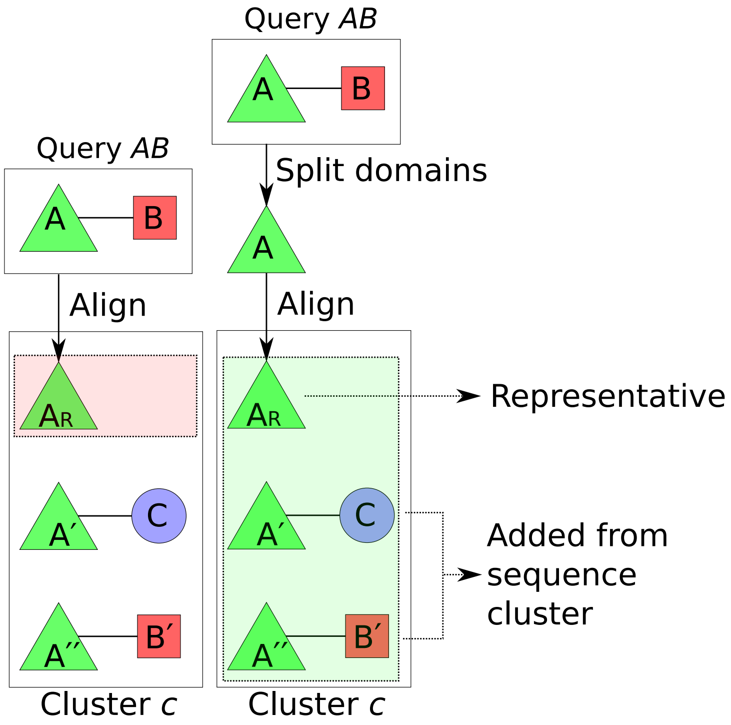

(a) Without splitting domains.

(b) With split domains.

Figure 3: Alignment of a multi-domain query AB against cluster c. The query protein is first aligned against the cluster representative A_R. If the alignment score is greater than the TM-score threshold, AB will be aligned against the remaining members of c.
